# Subtle Morality-Related Cues Promote Honest Behavior in Adolescents: Evidence from Chinese Middle School Students

**DOI:** 10.3390/bs16040587

**Published:** 2026-04-15

**Authors:** Tuo Zeng, Xinyi Tan, Zixin Yin, Kaixuan Huang, Jiawei Huang, Weijun Ma, Lei Mo, Sasa Zhao

**Affiliations:** 1 School of Educational Science, Jiaying University, Meizhou 514015, China; zengtuo@jyu.edu.cn (T.Z.); tzeng@jyu.edu.cn (J.H.); 2 Shatian Town Central Primary School, Dongguan 523000, China; tan_xyiyi@163.com; 3 Center for Studies of Psychological Application, South China Normal University, Guangzhou 510631, China; 20232921072@m.scnu.edu.cn; 4 Key Laboratory of Brain, Cognition and Education Sciences, Ministry of Education, South China Normal University, Guangzhou 510631, China; 5 School of Eco-Environment Technology, Guangdong Industry Polytechnic University, Guangzhou 510300, China; 2021090020@gdipu.edu.cn; 6 School of Psychology and Cognitive Science, East China Normal University, Shanghai 200062, China; wjma@psy.ecnu.edu.cn

**Keywords:** honest behavior, adolescent, verbal priming, visual priming

## Abstract

Honesty is essential for both individual development and the functioning of society. Although prior research has identified various factors that shape honest behavior, relatively little is known about whether adolescents’ honesty can be influenced by subtle morality-related cues, particularly among adolescents. The present study investigated whether exposure to verbal and visual morality-related cues would increase honest behavior in middle school students. Two behavioral experiments were conducted, each with 120 middle school students (aged 13–18) as participants. In Experiment 1, participants completed a Chinese idiom -unscrambling task with either the ethics-related or neutral characters. In Experiment 2, participants completed a visual cuing task involving either moral exemplar images or neutral images. In both experiments, honest behaviors were assessed via self-reported outcomes in a computerized coin-tossing task. Across both experiments, participants primed with morality-related words (Experiment 1) or moral exemplars (Experiment 2) demonstrated significantly more honest behavior in the coin toss task than those in the control group. These findings suggest that subtle verbal and visual morality-related cues can increase honest behavior in adolescents. The present study provides behavioral evidence that morality-related cues may shape honesty-related responding in adolescence and offers practical implications for promoting moral development through subtle contextual influences.

## 1. Introduction

Honesty refers to the tendency to convey truthful information even in the face of temptation (Greene & Paxton, 2009; Le et al., 2022; Cooper et al., 2023), and is also typically reflected in the voluntary inhibition of opportunistic self-serving behavior (Cohn et al., 2019; Zickfeld et al., 2025). Honesty is widely recognized as a foundational moral virtue, essential for individual character and the functioning of social systems (Le et al., 2022; Derfler-Rozin & Park, 2022; Cooper et al., 2023). Given its importance, scholars have long explored the psychological mechanisms and contextual factors influencing honest behavior (Dickinson & Masclet, 2023; Loe & Weeks, 2000; Reynolds & Ceranic, 2007; Pashler et al., 2013; L. Zhao et al., 2024; Puntiroli et al., 2026). However, existing research primarily focuses on relatively direct and explicit routes for promoting honesty, such as honesty reminders, rules, and incentive structures, while comparatively less attention has been paid to whether honesty may also be shaped by implicit or more subtle, cue-based influences embedded in the environment, particularly in adolescents (Hertwig & Mazar, 2022; Pruckner & Sausgruber, 2013; L. Zhao et al., 2024; Zickfeld et al., 2025). Based on this, the present study adopts two priming paradigms (one using morality-related verbal cues and the other using morality-related visual cues) to examine whether subtle morality cues influence honest behavior in middle school students.

According to theories of implicit cognition, moral concepts are represented in the mind as schemas or associative networks that can be automatically activated by relevant environmental cues, thereby shaping subsequent judgments and behavior outside of conscious awareness (Bargh, 2006; Gerlach et al., 2019; Lee et al., 2014; Talwar et al., 2016; Welsh & Ordóñez, 2014; but see J. Zhao et al., 2019 for null findings). For example, completing a moral sentence–unscrambling task has been shown to reduce cheating in adults (Welsh & Ordóñez, 2014), and listening to stories that highlight the positive value of honesty can significantly increase honesty among children (Talwar et al., 2016). Taken together, these findings suggest that moral priming in a subtle way may constitute an effective route for shaping honest behavior.

In addition, the existing literature still has two important limitations. First, prior work has focused primarily on adults or children, with adolescents—a critical developmental group—remaining largely unexplored (Derfler-Rozin & Park, 2022; Hertwig & Mazar, 2022; Puntiroli et al., 2026; L. Zhao et al., 2024). Dishonest behavior is relatively prevalent during adolescence, and effective early interventions can have enduring consequences for long-term moral development (Guo et al., 2023). Moreover, adolescence marks a pivotal period in which moral values shift from compliance with external norms toward the integration of internal self-standards (Blakemore & Mills, 2014; Crone & Fuligni, 2020; Killen & Smetana, 2023). During this transition, adolescents’ need for autonomy increases; thus, when explicit interventions are experienced as controlling or didactic, they may elicit psychological reactance and resistance (Daddis, 2011; Beyers et al., 2025). Accordingly, compared with approaches that rely on effortful self-control, implicit or subtle methods that do not require sustained conscious engagement may better fit adolescents’ developmental characteristics and yield more stable effects (Heiphetz, 2019; Bornstein et al., 2022). Second, implicit priming paradigms have been relatively narrow: Most prior studies have relied on semantic or narrative materials, such as sentence unscrambling tasks and moral stories, whereas more concrete, intuitive, and readily implementable visual cues (e.g., images) have received far less attention (Dai et al., 2023; Welsh & Ordóñez, 2014; Talwar et al., 2016; J. Zhao et al., 2019). Recent evidence suggests that exposure to superhero images as moral exemplars can increase prosocial intentions and helping behavior (Van Tongeren et al., 2018), highlighting the potential value of image-based moral cues.

Accordingly, the present study examined whether two types of morality-related cues (verbal cues and visual exemplar cues) would promote honest behavior among middle school students.

**H1.**  *Both morality-related cues (moral idiom unscrambling and moral exemplar images) would elicit increased honest behavior.*

To test this hypothesis, we designed two experiments using different priming tasks: a verbal priming task (Experiment 1) and a new visual priming task (Experiment 2). Given the uncertainty about the existence of this priming effect, we maximized the manipulation of moral concepts in Experiment 1 using a Chinese idiom-unscrambling priming task. Specifically, participants were presented with scrambled Chinese characters that could be rearranged into familiar idioms that emphasize integrity (e.g., “A gentleman’s word is his bond”, “君子一言，驷马难追” in Chinese). This task has been shown to be effective in inspiring honest behaviour in adults (Welsh & Ordóñez, 2014). In Experiment 2, we introduced a novel visual priming task, where participants were exposed to well-known moral figures from Chinese culture (e.g., Confucius, Lei Feng). Adolescents who are familiar with these figures and expressions may be particularly susceptible to these types of cue effects. In both studies, participants’ honesty was assessed using a self-reported computerized coin-flip task, a method widely used to measure honest behavior.

## 2. Experiment 1

### 2.1. Purpose

A Chinese idiom-unscrambling priming task was employed to investigate how subtle morality-related verbal cues promote honest behavior among Chinese middle school students.

### 2.2. Method

#### 2.2.1. Participants

A priori power analysis was conducted using G*Power 3.1 (Faul et al., 2007; Peer et al., 2024) to determine the required sample size. Assuming a Cohen’s d effect size slightly above the medium level at 0.55, with an alpha level of 0.05 and a statistical power of 0.8, the required sample size for an independent-samples *t*-test was calculated to be 53 participants per group, totaling 106. To ensure sufficient statistical power, we recruited 120 junior and senior high school students (balanced by gender) from a secondary school in Meizhou, Guangdong Province. Informed consent was obtained from parents or legal guardians and from all the students prior to participation. Ethical approval for the study was granted by the Scientific Research Ethics Committee of the South China Normal University (approval code: SCNU-PSY-2025-364). All participants had normal or corrected-to-normal vision, no color blindness or color weakness, no history of psychiatric disorders, and were all right-handed. Prior to the start of the experiment, participants were randomly assigned to two groups (Experimental vs. Control). At the end of the experiment, participants completed a post-experimental debriefing to assess awareness of the study purpose. Participants were not informed of the purpose before or during the task. Responses were coded as indicating awareness when participants explicitly expressed the possible link between the experimental manipulation and honesty-related behavior. Based on this criterion, participants who clearly guessed the study purpose were excluded from the primary analyses. Four students who guessed the purpose of the experiment were excluded from the analysis. The reported results are based on the remaining 116 valid participants: 57 in the experimental group (mean age = 15.93 years, SD = 1.40, range = 13–18) and 59 in the control group (mean age = 15.08 years, SD = 1.88, range = 13–18). As a robustness check, all main analyses were repeated with these participants retained, and the overall pattern of results remained unchanged. Participants received a small compensation for their participation at the end of the experiment.

#### 2.2.2. Materials

(1) Chinese idioms

Thirty idioms with moral meanings were selected from the Xinhua Chinese Dictionary. These idioms were randomly paired into 15 pairs. Then, the characters in each pair were randomly arranged to form 15 sets of moral idiom unscrambling materials. For example, one of the pairs is “君子一言，驷马难追” (A gentleman’s word is his bond), which was randomly rearranged as “驷、子、言、君、难、马、一、追”.

In addition, 150 students were recruited from a specific middle school (not involved in Experiment 1 and Experiment 2) and were asked to rate the difficulty of these 15 sets of materials as “difficult,” “moderate,” or “easy.” Based on the ratings, 10 sets were selected where over 90% of the students rated them as moderate or easy. These 10 sets were used as the final experimental materials.

The selection and creation of idiom unscrambling materials with neutral meaning followed the same method as above. However, these idioms have no connection to honesty or moral values. Thirty idioms with neutral meanings were selected, such as the pair “无声无息，水落石出” (Silent, like water revealing the stones). These were randomly rearranged as “水、无、声、落、出、无、石、息”. After rating the difficulty of these 15 sets of word formations, 10 sets were selected where over 90% of the students rated them as moderate or easy. These 10 sets were used as the official experimental materials.

(2) Emotion Assessment

The Positive and Negative Affect Schedule (PANAS) (Watson et al., 1988) was used to assess the mood. Redundant adjectives were removed, resulting in five positive emotion adjectives (happy, joyful, excited, delighted, and cheerful) and five negative emotion adjectives (sad, angry, afraid, nervous, and upset). Participants rated the extent to which they were currently experiencing each emotion using a 5-point Likert scale (1 = very slightly or not at all; 5 = extremely). The Cronbach’s alpha coefficients for the positive affect subscale and the negative affect subscale are 0.87 and 0.84, respectively.

(3) Honest Behavior Assessment: Coin Toss Game

The coin toss task was programmed using Python 3.6 and administered on a computer. Participants were seated in front of a screen and instructed to follow on-screen prompts using a mouse.

#### 2.2.3. Experimental Design and Procedure

Experiment 1 employed a between-subjects design with a single factor, where the independent variable was the priming word (morality-related words vs. neutral words), and the dependent variable was the participants’ honesty score in the coin-tossing task.

**Figure 1:**
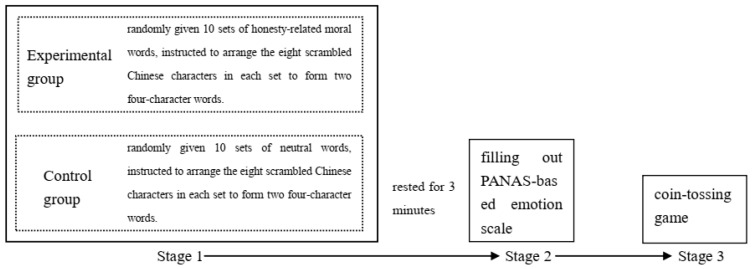
Flow chart of Lab 1.

The entire process was divided into three stages (see Figure 1).

Stage 1 involved the word formation priming task. Since the speed at which participants input Chinese characters could affect the priming effect, Experiment 1 used a paper-and-pencil method for responses. Participants in the experimental group were randomly given 10 sets of morality-related words, while participants in the control group were given 10 sets of neutral words. They were instructed to arrange the eight scrambled Chinese characters in each set to form two four-character words. No feedback was provided during the process. All participants were able to complete the word formation task within 4 to 6 min, with a correct rate of over 90%. The vast majority of participants performed the task correctly, with only a few participants making one or two errors. After completing the word formation task, participants rested for 3 min.

Stage 2 involved participants filling out the PANAS-based emotion scale.

Stage 3 was the coin-tossing game.

**Figure 2:**
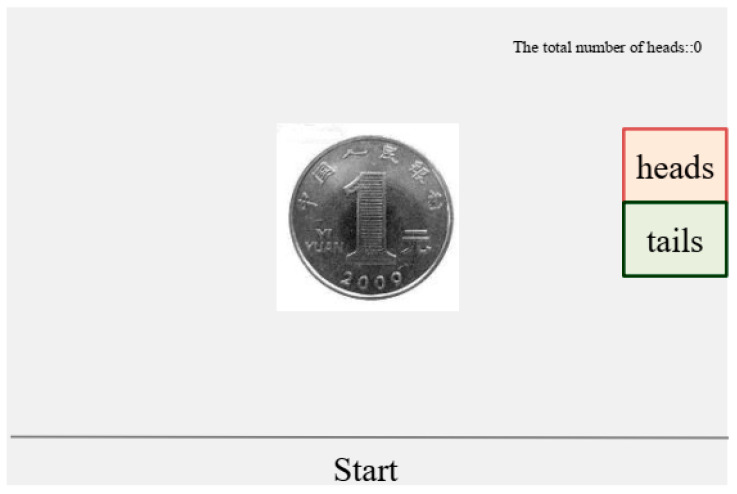
Coin Toss Game Interface.

Participants were informed that the purpose of the task was to practice spinning and stopping a coin by the mouse, and to experience the role of luck in determining whether the coin landed on heads. The coin, which had two sides (heads and tails), was initially displayed at the center of the screen, with a “Start” button positioned below it (see Figure 2). Participants clicked “Start” to initiate the continuous rotation of the coin and clicked “Stop” to halt it. Upon stopping, the coin displayed either heads or tails. On the right side of the screen, two buttons labeled “Heads” and “Tails” were provided for recording the outcome. Participants were instructed to click “Heads” if the coin landed heads up, and “Tails” if it landed tails up. The computer automatically recorded the total number of heads for each participant, with a monetary reward of 2 yuan awarded for each recorded head. Participants were assured that their coin toss process and results were completely private and could not be viewed by anyone else.

The outcome of the game was self-reported by participants. Although participants were free to misreport without detection, the program was pre-set to display a “heads” result only four times. Therefore, if a participant reported exactly four heads, they were considered fully honest. Any number above four indicated dishonesty, with the maximum possible number of false reports being six.

The experiment included a “warm-up” session followed by a “formal” session. The formal session consisted of 10 tosses, with participants earning more money for reporting more heads (reward = number of heads × 2 yuan). Before beginning the formal session, participants completed five warm-up trials.

Although participants were technically eligible for monetary rewards based on their reported number of “heads,” ethical considerations were taken into account. Since the value system and moral judgment of middle school students are still developing, providing higher rewards for dishonesty might foster unethical behavior and negatively influence their growth. Therefore, at the end of the experiment, during compensation distribution, each participant received the following explanation from the researchers:

“*Dear student, thank you for your participation and support! In this experiment, some students obtained more ‘heads’ than others. However, we encourage mutual support and sharing. Given the limited budget, we will distribute the rewards equally among all participants. Thank you for your understanding.*”

Finally, each participant was required to provide a written response indicating whether they had guessed the purpose of the experiment. If the response was “Yes,” they were asked to state what they believed the purpose was.

### 2.3. Analyses and Results

The honesty score was determined based on the number of instances of dishonesty, with scores assigned in a reverse order. Specifically, participants who lied 0 to 6 times received honesty scores of 7, 6, 5, 4, 3, 2, and 1, respectively.

#### 2.3.1. Comparison of Honesty Scores Among Participants Initiated by Different Character Images

To determine whether honesty differed between participants primed with morality-related versus neutral word-formation materials, we conducted an independent-samples *t*-test comparing honesty scores in the experimental and control groups. Results showed that the experimental group exhibited significantly higher honesty scores than the control group. As shown in Table 1, subjects in the experimental group achieved significantly higher honesty scores compared to subjects in the control group (*t* = 4.38, *p* < 0.001, Cohen’s d = 0.79).

#### 2.3.2. Comparison of Emotional States After Word Formation Priming

To examine whether differences in honest behavior between the experimental and control groups were influenced by their emotional states, an independent-samples *t*-test was conducted to compare the emotions of participants in both groups after completing the word formation priming task. The results are presented in Table 2. As shown in Table 2, there was no significant difference in positive emotional states between the two groups (*t* = −0.92, *p* = 0.36), and there was also no significant difference in negative emotional states (*t* = −1.26, *p* = 0. 21).

Since there were no significant differences in emotional states between the experimental group (morality-related group) and the control group (neutral group), the observed effects are not explained by differences in general affective states (positive or negative) as measured by PANAS.

## 3. Experiment 2

### 3.1. Purpose

A visual priming paradigm was employed to investigate the subtle cueing effect of moral exemplar images (vs. neutral images) on honest behavior among Chinese middle school students.

### 3.2. Method

#### 3.2.1. Participants

A priori power analysis was conducted using G*Power 3.1 (Faul et al., 2007; Peer et al., 2024) to determine the required sample size. Assuming a Cohen’s d effect size slightly above the medium level at 0.55, with an alpha level of 0.05 and a statistical power of 0.8, the required sample size for an independent-samples *t*-test was calculated to be 53 participants per group, totaling 106. To ensure sufficient statistical power, we recruited 120 junior and senior high school students (balanced by gender) from another secondary school in Meizhou, Guangdong Province. Informed consent was obtained from parents or legal guardians, and from all the students prior to participation. Ethical approval for the study was granted by the Scientific Research Ethics Committee of the South China Normal University (approval code: SCNU-PSY-2025-364). All participants had normal or corrected-to-normal vision, no color blindness or color weakness, no history of psychiatric disorders, and were all right-handed. Participants were randomly assigned to two groups. Three students who guessed the purpose of the experiment were excluded, resulting in 117 valid participants: 58 in the experimental group (mean age = 15.76 years, SD = 1.17, range = 13–18) and 59 in the control group (mean age = 15.59 years, SD = 1.35, range = 13–18). Participants received a small compensation for their participation at the end of the experiment.

Experiment 2 adopted a single-factor between-subjects design. The independent variable was the type of visual priming image (moral exemplar vs. neutral individual), and the dependent variable was the honesty score based on self-reports in the coin toss game. Participants were assigned randomly to the experimental group viewing images of Confucius and Lei Feng or the control group viewing two neutral individual images.

#### 3.2.2. Materials

(1) Visual Images for Priming

**Figure 3:**
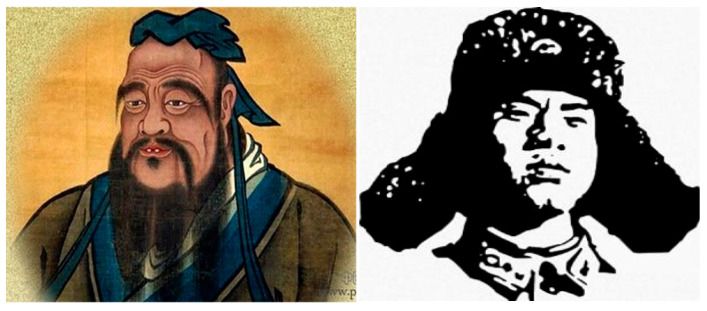
Moral Exemplar Images.

Two widely recognized moral exemplars—Confucius and Lei Feng—were selected as the moral exemplar images, with one image each, standardized to 15 × 13 cm^2^. These served as the visual priming stimuli for the experimental group (see Figure 3).

**Figure 4:**
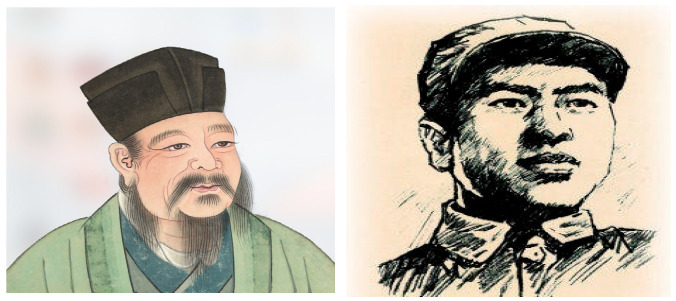
Neutral Individual Images.

Two neutral images of individuals resembling Confucius and Lei Feng in era, age, gender, and appearance were identified using systematic online searches. These images, also sized 15 × 13 cm^2^, served as visual priming stimuli for the control group (see Figure 4).

(2) Emotion Measurement
  The emotion was measured using the same scale as in experiment 1.

(3) Honest Behavior Assessment: Coin Toss Game
  The coin toss game for honest behavior assessment was the same as experiment 1.

#### 3.2.3. Experimental Design and Procedure

**Figure 5:**
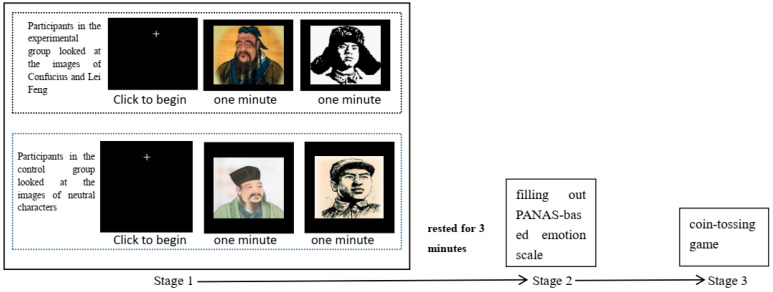
Flow chart of Lab 2.

The experiment consisted of three stages (see Figure 5).

Stage 1: Visual Priming on Computer

Participants in the experimental group looked at the images of Confucius and Lei Feng, while the participants in the control group looked at two pictures of neutral characters. Participants sat in front of a computer and were told that two images of individuals would appear on the screen, each displayed for one minute. They were instructed to carefully attend to and observe the features of each image.

Stage 2: Completion of the Emotion Assessment Scale

Participants filled out the PANAS immediately after the visual priming task.

Stage 3: Self-Reported Coin Toss Game on Computer

The same coin toss game was conducted as Experiment 1. At the end of the experiment, the researchers also had to explain to each participant how much they were paid on average. The participants were asked to answer the two questions in writing about whether they guessed the purpose of the experiment.

### 3.3. Analyses and Results

In Experiment 2, the participants’ honesty score in the coin toss game was the same as that in Experiment 1.

#### 3.3.1. Comparison of Honesty Scores Among Participants Initiated by Different Character Images

An independent samples *t*-test was conducted to compare whether there was a significant difference in honesty scores between participants in the experimental group and those in the control group during the coin-tossing game. As shown in Table 3, participants in the experimental group achieved significantly higher honesty scores in the coin-tossing game compared to participants in the control group who were primed with neutral character images (*t* = 4.92, *p* < 0.001, Cohen’s d = 0.91).

#### 3.3.2. Comparison of Emotional States After Visual Priming

To examine whether differences in honest behavior between the experimental and control groups were influenced by their emotional states, an independent-samples *t* test was conducted to compare the emotion of participants in both groups after completing visual priming task. The results are presented in Table 4. As shown in Table 4, there was no significant difference in positive emotional states between the two groups (*t* = 1.17, *p* = 0.25), and there was also no significant difference in negative emotional states (*t* = 0.40, *p* = 0.69).

Since there were no significant differences in emotional states between the experimental group (moral exemplar image priming group) and the control group (neutral character image priming group), the observed effects are not explained by differences in general affective states (positive or negative) as measured by PANAS.

## 4. Discussion

The present study aimed to investigate the influence of subtle morality-related cues on honest behavior among Chinese middle school students. Specifically, we examined the effects of subtle verbal cues (moral idiom unscrambling) and visual exemplar cues (exposure to moral figures) on participants’ honesty in a computerized coin-flip task. The results demonstrate that exposure to subtle morality-related cues significantly increased honest behavior among adolescents.

In Experiment 1, participants who unscrambled Chinese characters into integrity-emphasizing idioms exhibited higher levels of honesty compared to those in the control condition. This finding is consistent with previous research on adults and children, which has shown that exposure to an ethical sentence-unscrambling task or moral stories can inspire honest behavior (Dai et al., 2023; Welsh & Ordóñez, 2014; Talwar et al., 2016). Our study extends this effect to adolescents, suggesting that verbal cues related to morality can effectively promote honesty in this age group. This is particularly important given the developmental changes and increased susceptibility to social influences during adolescence (Blakemore & Mills, 2014; Crone & Fuligni, 2020; Killen & Smetana, 2023).

Experiment 2 introduced a novel visual priming task, in which participants were exposed to images of well-known moral figures from Chinese culture, such as Confucius and Lei Feng. The results showed that participants in the moral exemplar condition displayed significantly more honest behavior than those in the control condition. This finding demonstrates the potential of visual cues, particularly those depicting moral exemplars, to influence adolescents’ honesty. Recent research has highlighted the role of moral exemplars in moral development (Dykhuis et al., 2025; Maranges et al., 2024), and our study adds evidence in adolescents.

One plausible possibility is that morality-related cues served as reminders of broader moral standards, thereby making these standards more accessible at the moment of choice and increasing the likelihood of honest behavior (Mazar et al., 2008; Leach & Iyer, 2024). This interpretation aligns with recent meta-analytic evidence showing that moral reminders and honesty commitments can effectively reduce dishonest behavior by increasing the salience of moral values (Zickfeld et al., 2025).

Compared to previous research, our study provides novel insights into the effects of morality-related subtle cues on honest behavior in adolescents. While prior studies have primarily focused on adults or children (Hertwig & Mazar, 2022; Puntiroli et al., 2026; L. Zhao et al., 2024), our findings demonstrate that these effects extend to the critical developmental period of adolescence. Moreover, the role of visual moral exemplar cues in honest behavior extends and complements existing research focused on moral semantic cues (Hertwig & Mazar, 2022).

The present study contributes to the growing body of literature on the factors influencing honest behavior in adolescents (Evans & Talwar, 2024; Guo et al., 2023). Our findings suggest that subtle cues related to morality can have a significant impact on adolescents’ honest behavior. This has important implications for educators and policymakers seeking to foster integrity and ethical behavior among young people. Incorporating morality-related cues into educational materials and environments may be an effective strategy for promoting honesty and reducing dishonest behavior (Evans & Talwar, 2024).

However, it is important to acknowledge the limitations of the current study. First, given that the experiments were conducted exclusively with Chinese middle school students, the generalizability of the findings to other age groups (e.g., elementary or university students) and cultural contexts remains to be further investigated. Future research could benefit from incorporating diverse cultural backgrounds and age groups to further validate the observed effect (Cohn et al., 2019; Gächter & Schulz, 2016; Keller & Kiss, 2025). Second, our research design captures only the immediate, short-term effects of moral cues. It remains unknown whether such priming effects of subtle cues can persist or accumulate over time with repeated application to foster enduring moral behavior. To address this gap, future studies could employ repeated-exposure paradigms to test whether the effect of a moral cue decreases, stabilizes, or even strengthens over time. Last, while our study focuses on the effects of subtle moral cues on honest behavior, we recognize the value of integrating a dual-process perspective that examines whether honesty is driven by intuitive versus deliberative cognitive processes. Future research could explore the intersection of these approaches by investigating whether the effectiveness of subtle moral cues varies as a function of cognitive processing mode—for example, under time pressure versus time delay conditions (Capraro, 2024; Margoni et al., 2025; Köbis et al., 2019). Such work would yield deeper insight into how external subtle morality cues shape honest behavior across different processing modes.

## 5. Conclusions

The present study hypothesized that two types of morality-related cues, verbal cues (moral idiom unscrambling) and visual exemplar cues (moral exemplar images), would elicit increased honest behavior among Chinese middle school students. The results support this hypothesis, as both types of cues were shown to significantly enhance honest behavior when compared to the control condition. These findings contribute to our understanding of the factors influencing adolescents’ moral behavior and have important implications for the design of interventions to promote honesty and integrity. These findings offer evidence for the effectiveness of nudging techniques in promoting honesty and suggest practical applications for fostering moral behavior in educational contexts.

## Figures and Tables

**Table 1:** Comparison of honesty scores between experimental and control groups.

Group	*n*	*M* ± *SD*	*t*	Cohen’s d
Experimental	57	5.51 ± 0.71	4.38 ***	0.79
Control	59	4.68 ± 1.27		

*** *p* < 0.001.

**Table 2:** Comparison of emotion scores between experimental and control group.

Group	*n*	Positive Emotion		Negative Emotion	
		*M* ± *SD*	*t*	*M* ± *SD*	*t*
Experimental	57	9.56 ± 4.86	−0.92	6.12 ± 1.30	−1.26
Control	59	10.42 ± 5.24		6.62 ± 2.79	

**Table 3:** Comparison of honesty scores between experimental and control groups.

Group	*n*	*M* ± *SD*	*t*	Cohen’s d
Experimental	58	5.83 ± 0.42	4.92 ***	0.91
Control	59	4.81 ± 1.53		

*** *p* < 0.001.

**Table 4:** Comparison of emotion scores between experimental and control groups.

Group	*n*	Positive Emotion		Negative Emotion	
		*M* ± *SD*	*t*	*M* ± *SD*	*t*
Experimental	58	10.97 ± 5.65	1.17	6.78 ± 2.80	0.40
Control	59	9.80 ± 5.18		6.59 ± 2.09	

## Data Availability

The original data presented in the study are openly available in OSF at https://osf.io/d67hj/overview, accessed on 1 February 2026.
